# Energy Requirement and Food Intake Behaviour in Young Adult Intact Male Cats with and without Predisposition to Overweight

**DOI:** 10.1100/2012/509854

**Published:** 2012-05-01

**Authors:** Brigitta Wichert, Julia Trossen, Daniel Uebelhart, Marcel Wanner, Sonja Hartnack

**Affiliations:** ^1^Institute of Animal Nutrition, Vetsuisse Faculty, University of Zurich, Winterthurerstraße 260, 8057 Zurich, Switzerland; ^2^Department of Rheumatology and Institute of Physical Medicine, University Hospital Zurich, Gloriastraße 25, 8091 Zurich, Switzerland; ^3^Section of Epidemiology, Vetsuisse Faculty, University of Zurich, Winterthurerstraße 270, 8057 Zurich, Switzerland

## Abstract

Obesity is a common problem in cats. In the experimental cat family of the institute of animal nutrition besides a “normal” lean phenotype, cats with predisposition to an overweight phenotype are present. To investigate energy requirements and food intake behaviour of intact male cats of different phenotypes, six “normal” lean cats (GL) and six cats disposed to overweight (GO) were used. At the beginning of the experiment, all cats had an ideal body condition score of 5. To reach this the GO cats had to pass a weight-loss program. Energy requirements of the cats were determined using respiration chambers, whereas the amount and frequency of food intake was measured with a feeding station recording the data automatically. Energy requirement at weight constancy of the GO cats was even on fat-free mass (FFM) significantly (*P* = 0.02) lower (162.6 kJ/kg FFM/d) than that of the “normal” lean cats (246 kJ/kg FFM/d). The GO cats also showed a higher food intake 34.5 ± 1.5 g dry matter/kg body weight^0.67^ compared to the GL cats (24.0 ± 2.1 g dry matter/kg body weight^0.67^)(*P* = 0.001). In conclusion quantifiable differences in food intake and behaviour in cats predisposed to overweight compared to “normal” lean cats were found.

## 1. Introduction

Obesity is an increasing problem in cats. In recent studies, the prevalence of feline obesity in different countries has ranged from 6% to 52% [1, 2]. Obesity is the result of a higher energy intake in comparison to the energy requirement. This can be caused by an increased energy intake, decreased energy expenditure, or both. Multiple factors like castration, highly palatable food, cats keeping indoors, and genetic factors are related to obesity [2–4]. In several human and rodent studies, a genetic background was supported [5–7]. Recently also in cats a genetic association with overweight has been described in the experimental cat population of the Institute of Animal Nutrition of the Vetsuisse Faculty of Zurich [8]. However, to our knowledge, nothing is known about energy expenditure or food intake behaviour of intact cats developing overweight. An increase in food intake can be due to a higher meal frequency or a higher food intake per meal. The cat as a hunter of small prey animals has to eat about twelve small prey animals, corresponding to twelve meals in 24 hours [9]. A similar meal frequency of about 12–20 meals per day was observed in the studies of Mugford and Thorne [10], Kane et al. [11], and Peachey and Harper [12]. However, Horwitz et al. [13] described that meal size was influenced by daytime as well as palatability and protein content of food. In contrast to this, the speed of food intake was more connected to food structure. 

The aim of the present study was to quantify the difference in energy expenditure or food intake behaviour in intact male cats with and without predisposition to overweight.

## 2. Material and Methods

### 2.1. Cats

The study population consisted of six intact male cats displaying a phenotype lean (group GL) and six intact male cats with a phenotype overweight (group GO) aged 1.3 ± 0.06 years. Classification into phenotype lean/overweight was based on body condition score (BCS) measured at month eight according to Laflamme [14]. All cats were reared under the same conditions, clinically healthy, and intact male European Shorthair cats. They originated from a larger breeding project with a focus on segregation of lean and overweight phenotypes in the cat-breeding family of the Institute of Animal Nutrition at the Vetsuisse Faculty, Zurich [8]. Ethical approval for animal experimentation was obtained from the Swiss veterinary authorities (license number 180/2009).

### 2.2. Study Design

**Figure 1:**
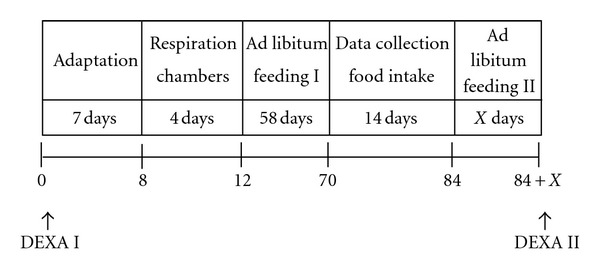
Experimental study design of the five experimental phases including measurements in the respiration chambers, measurements of food intake as well as dual energy X-ray absorptiometry measurements.

The experimental study design comprised five different phases (Figure 1). Before starting the experiment, all cats of group GO passed through a weight loss program until reaching a BCS of 5. After that the cats of group GO were fed to maintain a constant body weight (BW) for at least 4 weeks. The cats of group GL were fed ad libitum during the whole experiment.

#### 2.2.1. Adaptation Period (Duration 7 Days)

The cats of group GO were fed to maintain a constant bodyweight (BW). During the adaptation period, cats were adapted to a commercial dry cat food (Table 1) and special cat toilettes allowing for separate collection of faeces and urine [15].

#### 2.2.2. Respiration Chamber Period (Duration 5 Days)

Cats were kept individually for 22.5 hours per day in the respiration chambers (1.4 m × 1.0 m × 0.9 m). After one day for adaptation, on a daily basis, O_2_ consumption and CO_2_ production were determined with reference to body weight, body weight^0.67^, and fat-free mass [16]. On a daily basis, faeces and urine were collected separately, and food intake was measured. From these data nitrogen, carbon and energy balances were calculated. 

An equalized energy balance at weight constancy is named energy requirement in the text although energy consumption in respiration chambers mostly is lower compared to that under usual living conditions, as activity is limited.

#### 2.2.3. Ad Libitum Feeding Phase I (Duration 58 Days)

During this period cats were kept in groups of two cats each in an indoor-outdoor enclosure.

#### 2.2.4. Automatic Food Station Phase (Duration 14 Days)

**Figure 2:**
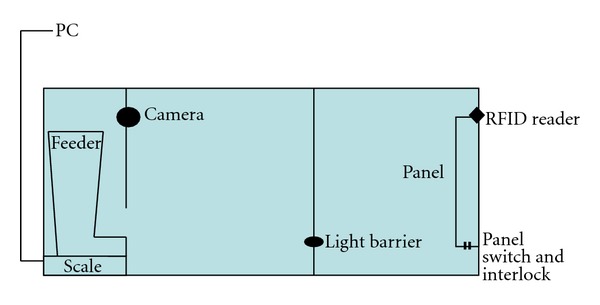
Design of the automatically cat food station: documenting food intake per meal, meal frequency, visits of the station without eating, and the time of entering the station. The cats were recognized by their implanted microchip.

During this phase, food intake per meal, frequency of meals, visits of the station without eating, and the time of entering the station were recorded. The intake of metabolisable energy (ME) was calculated using the ME-content of the dry food determined during the respiration measurement. Additionally, each action of the cats was documented using a camera. The automatic feeding station (Figure 2) recognized the cats by their implanted chip and assured that there was only one cat each inside the station with help of a light barrier that recognized movements in the feeding station. For the function of the feeding station as well as the documentation of the data, special software was developed (Gruber Informatik AG, Bergdietikon, Switzerland).

#### 2.2.5. Ad Libitum Feeding Phase II (Duration Variable; Min 4 Weeks, Max 27 Weeks)

During this period two cats were kept together in an indoor-outdoor enclosure and fed until a constant body weight was obtained for at least four weeks.

### 2.3. Measurements and Analysis

#### 2.3.1. Body Weight and Body Fat

During the measurements in the respiration chamber, the cats were weighed daily, during other phases weekly. As shown in Figure 1, before respiration measurements and at the end of the second ad libitum feeding phase after four weeks of weight constancy, body fat content and lean body mass (=fat-free mass (FFM)) were determined by dual X-ray absorptiometry (DEXA). For the DEXA measurement in the sternal recumbency, a Hologic QDR 4500 Discovery machine (Bedford, MA, USA) was used. QDR System software Version 12.4 and the Infant Whole Body scan type were used. The machine's lower body fat-measuring limit was approximately 4%. For data analyses, the global mode (includes the whole body with extremities and head) was used. The cats were sedated with medetomidine 0.05 mg/kg BW and butorphanol 0.2 mg/kg BW.

#### 2.3.2. Statistical Analyses

The objective of the statistical analysis was to test the hypothesis of difference in “normal” lean cats and cats predisposed to overweight. For comparisons between two groups at the same time point or two time points within the same group, non-parametric tests (Wilcoxon rank sum and Wilcoxon signed rank) were performed with the software NCSS 2007 version 7.1.20 (Kaysville, Utah, USA). Statistical significance was set at *P* ≤ 0.05. To estimate a potential effect of phenotype overweight versus lean during repeated measurements in the respiration chamber, linear and linear mixed effects models were used with the software R version 2.11.1 [17] and the packages nlme [18] and lmtest [19]. *P* values for linear models were derived from likelihood ratio tests. In order to account for potential correlation of measurements within cats, cat was used as random effect. Model selection was based on (AIC Akaike's information criterion) with a lower AIC indicating a better model fit and visual checking of the random effects residuals. Highly influential or leverage data points were determined using Cook's distance. Results are given in marginal means of effect sizes and their corresponding standard errors (S.E.).

## 3. Results

### 3.1. Body Weight and Fat Content

At the beginning of the adaptation period, there was no statistical difference in body weight between groups, but in body fat content (fat mass in g *P* = 0.02, fat mass in % *P* = 0.03) with group GO having a lower fat content (Table 2). Statistical differences between both groups were found at the end of the second ad libitum phase, with the group GO having a statistically significant higher body weight (*P* = 0.013) and body fat content (fat mass in g *P* = 0.005, fat mass in % *P* = 0.005) compared to group GL. Whereas the measurements in group GL did not differ significantly in between the first and the second measurement, in group GO statistical significant differences were found (body weight *P* = 0.02, body fat mass in g *P* = 0.005, body fat mass in % *P* = 0.005).

### 3.2. Respiration Measurements

Using a linear mixed model approach, significant differences in O_2_ consumption as well as CO_2_ production between both phenotypes, with phenotype GO having lower values were found (Table 3).

Two highly influential data points, which corresponded to the first measurements of O_2_ consumption in each group and were assumed to be atypical, have been removed.

Based on pooled samples (four sampling days taken together for each cat), energy requirements at weight constancy for both groups was estimated with a linear regression approach with retained energy as independent and ME intake as dependent variable, additionally separately for each group. In this calculation the estimated intercept corresponds to the energy requirement at weight constancy of 246 (S.E. 22.6) kJ/BW FFM in group GL and 162.6 (S.E. 14.3) kJ/BW FFM in group GO. The estimated mean effect on energy value of eaten diet intake of group GO compared to GL was a reduction of 83.6 (S.E. 26.4) kJ/BW FFM (*P* = 0.022). The interaction between group effect and retained energy was significant (*P* = 0.01), indicating that the association between ME uptake and retained energy was significantly different between the two groups. The corresponding regression coefficients (indicating the increase in ME uptake per one unit increase in retained energy) were 0.73 (S.E. 0.14) for group GL and 1.2 (S.E. 0.12) for group GO. If ME uptake and retained energy were referred to BW^0.67^, energy requirement was calculated to be 351 (S.E. 34.2) kJ/BW^0.67^ in group GL and 261 (s.e. 29.8) kJ/BW^0.67^ in group GO. The interaction between group effect and retained energy was not significant. The corresponding regression coefficients were 0.9 (S.E. 0.15) for group GL and 1.25 (S.E. 0.17) for group GO.

### 3.3. Food Intake Measurements

During the ad libitum feeding using the automatic feeding station, the cats of the obese phenotype (GO) showed a significantly (*P* = 0.001) higher food intake than the cats of group GL. It corresponded to an ME intake of 422 ± 37 kJ/kg BW^0.67^ in group GL and 622 ± 27 kJ/kg BW^0.67^ in group GO. Also the meal size differed significantly between the two groups, but the stay in the cat food station did not (Table 4). The meal frequency with 9.5 ± 0.3 times/24 hours in group GL and 7.2 ± 0.2 times/24 hours in group GO did not differ significantly (*P* = 0.17).

## 4. Discussion

In this experimental study a significant association between phenotype overweight versus lean in the following measured variables became evident: body weight and body fat, O_2_ consumption, CO_2_ production, energy requirement, food intake in g and in energy value of eaten diet, and meal size, but not duration of stay or frequency. The cats of group GL showed a constant body weight during the whole study time, despite ad libitum feeding. In contrast, cats from group GO started gaining body weight soon after the beginning of ad libitum feeding. Despite similar BCS and body weight in both groups initially, group GO had a significantly lower body fat content than the animals of group GL. This could be due to a misinterpretation of abdominal skin as abdominal fat as this is one important criteria in the body condition scoring system after Laflamme [14]. At the end of the second ad libitum phase, after four weeks of weight constancy, only the cats with phenotype GO showed a significant increase in body fat, and they had a significantly higher body fat content than the cats of group GL. This difference supported the assumed different phenotypes [8] and was expected as a weight regain after a weight-loss program of overweight individuals is a known effect in pets [20, 21].

Although kept under similar conditions, with a similar potential for activity, the measurements taken in the respiration chamber indicate a significantly lower metabolic activity of the cats with phenotype GO compared to phenotype GL. Due to the availability of pooled samples to estimate energy requirements only one data point for each animal could be used in a linear regression approach. This potentially lowered the power to detect a significant difference between the two groups with regard to their energy requirements if referred to BW^0.67^. The estimated intercepts still indicate a potential higher energy requirement in group GL compared to group GO. Although not significant, the estimated regression coefficients, corresponding to the association between ME and retained energy, might indicate a different relationship between ME and retained energy in both groups. If the energy requirements were referred to fat-free body mass, a significant difference between both groups was found. This finding indicates that referring energy requirements to fat-free body mass might be more appropriate than to BW^0.67^. With regard to the significant difference in energy requirements between the cats of phenotype overweight and lean, it might have happened in this study design, that the weight-loss program affected the energy expenditure of the group GO, since energy restriction in cats and dogs can result in a decrease in relative energy expenditure [21–23]. The potential effect due to the weight-loss program in cats of phenotype GO might have been persisted during the following different phases explaining part of the differences found. A potential way to circumvent the potential influence of a weight-loss program would be to evaluate energy requirements earlier in life, before a distinct phenotype becomes evident (and a weight-loss program necessary to assure a similar BCS in both groups). Also a higher amount of less metabolically active fat tissue has been assumed to cause lower-energy requirements in overweight cats compared to their lean counterparts [24, 25]. This can be excluded, however, for the cats of the present study, since the calculation of energy requirements based on fat-free mass confirmed the observed differences between the cats of the different phenotypes. It can be speculated that the difference in energy requirements also based on FFM, the possibly different relationship between ME and retained energy, and in O_2_ consumption and CO_2_ production were influenced by differences in activity of the cats of the two groups. 

In the group GO, a significantly higher food intake either in g and in terms of energy value of diet and meal size became obvious during the measurements in the automatic cat feeding station. But duration of stay or frequency was not associated with phenotype.

Increased food intake and reduced energy expenditure in cats soon after castration has been described [20, 24]. The cats in the present study were intact males in their young adulthood originating from the same cat population and reared under similar conditions. Ad libitum access to feed led to an increase in body weight and a body condition score indicative of overweight within a couple of weeks in the group predisposed to overweight but not in the normal lean group (GL). In obesity mouse models, decreased energy expenditure and increased food intake have been associated with mutations in the leptin system [26]. Also in obesity of humans some mutations influencing the eating behaviour are known [5, 27–30].

In humans a disordered energy balance with decreased energy expenditure and increased appetite after a weight reduction program in comparison to “normal” lean humans was observed [31]. The data of the present study support the assumption that the feeling of satiety is affected in the cats of the GO group, predisposed to overweight, since food intake, but not meal frequency or stay was significantly different between both groups. Meal frequency of all cats of the present study was in agreement with the observed frequencies by Mugford and Thorne [10], Kane et al. [11], and Peachey and Harper [12]. Because of this homogenous behaviour, rank order as cause of the differences in food intake could be excluded. This was also documented by the fact that the program did not recognize one cat of each group more often without entering the cat food station at the end. It could be concluded that the cats higher in ranking did not block the entrance of the cat food station for the other cat for a longer time. Apparently all cats followed their natural eating behaviour.

In conclusion, in the present study, the differences in food intake and energy expenditure in young intact male cats with and without predisposition to overweight could be quantified. The results indicate that in the cats showing an overweight phenotype the regulation of food intake differs from that of the “normal” lean cats. To specify the influence of energy expenditure without any weight-loss program, further investigation at earlier age in cats of the named experimental cat family is needed.

## Figures and Tables

**Table 1:** Chemical composition (%) of the commercial dry cat food used during the whole experiment.

Ingredients	Content
Crude protein	32%
Crude fat	21%
Crude fiber	2%
Crude ash	6%
Nitrogen-free extracts	31%
Dry matter	92%
Gross energy	22.9 kJ

**Table 2:** Mean of body weight (BW), body fat mass (total (g), and (%)) fat-free body mass (total (g), and (%)) and body weight (g) of the cats of the groups GL (lean phenotype) and GO (overweight phenotype); DEXA I: first DEXA measurement before the adaptation period; DEXA II: second DEXA measurement at the end of the second phase of ad libitum feeding after four weeks of weight constancy.

	GL		GO	
	DEXA I	DEXA II	DEXA I	DEXA II
Body weight ± SE (g)	5063 ± 251^aA^	4992 ± 267^aA^	5036 ± 261^aA^	6487 ± 324^bB^
Fat mass ± SE (g)	380 ± 51^aA^	336 ± 81^aA^	199 ± 39^aB^	893 ± 94^bB^
Fat mass ± SE (%)	7 ± 1^aA^	7 ± 1^aA^	5 ± 1^aB^	14 ±1^bB^
Fat free mass ± SE (g)	4543.0 ± 214.4^aA^	4656.2 ± 196.9^aA^	4768.8 ± 262.2^aA^	5429.7 ± 251.6^bB^
Fat free mass ± SE (%)	89.8 ± 0.8^aA^	90.7 ± 1.4^a**A**^	92.0 ± 0.8^aB^	83.8 ± 1.0^b**B**^

Different letters show significant differences (*P* < 0.05), capital letters show group differences, whereas small letters show differences within one group.

**Table 3:** Differences between both phenotypes estimated with a linear mixed model approach (group means and standard errors of the estimated effect due to phenotype; GL: lean phenotype, GO: overweight phenotype).

	Phenotype GL	Phenotype GO	S.E.	*P* value
	mean	mean		
O_2_ L/kg BW^0.67^/d	19.67	17.31	1.05	0.026
O_2_ L/kg BW/d	11.71	10.26	0.64	0.035
O_2_ L/kg FFM/d	12.88	10.87	0.8	0.022
CO_2_ L/kg BW^0.67^/d	16.66	14.75	0.64	0.008
CO_2_ L/kg BW/d	9.59	8.74	0.4	0.046
CO_2_ L/kg FFM/d	10.57	9.25	0.46	0.01

**Table 4:** Mean food intake, duration of single stays, food intake/min of stay, meal size, and meal frequency in the cat food station of the cats of group GL (lean phenotype) and GO (overweight phenotype), different letters show significant differences (*P* < 0.05).

	Group GL	Group GO
Food intake mean ± SE (g DM/kg BW^0.67^/d)	24.0 ± 2.1^**A**^	34.5 ± 1.5**^B^**
Duration of stay mean ± SE (min/d)	3.3 ± 0.2^A^	4.4 ± 0.5^A^
Food intake mean ± SE g/min of stay	2.6 ± 0.1**^A^**	3.7 ± 0.1**^B^**
Meal Size mean ± SE (g/d)	8.6 ± 0.5**^A^**	16.6 ± 1.8**^B^**
Meal frequency mean ± SE/d	9.5 ± 0.3^A^	7.2 ± 0.2^A^
